# The Bidirectional Relationship between Posttraumatic Stress Symptoms and Social Support in a 9/11-Exposed Cohort: A Longitudinal Cross-Lagged Analysis

**DOI:** 10.3390/ijerph19052604

**Published:** 2022-02-24

**Authors:** Sze Yan Liu, Jiehui Li, Lydia F. Leon, Ralf Schwarzer, James E. Cone

**Affiliations:** 1 New York City Department of Health and Mental Hygiene, World Trade Center Health Registry, New York, NY 10279, USA; lius@montclair.edu (S.Y.L.); lleon1@health.nyc.gov (L.F.L.); jcone@health.nyc.gov (J.E.C.); 2 Department of Psychology, Freie University of Berlin, 14195 Berlin, Germany; ralf.schwarzer@fu-berlin.de; 3 Department of Psychology, SWPS University of Social Sciences and Humanities, 03-815 Warsaw, Poland

**Keywords:** posttraumatic stress disorder, 9/11 disaster, social support, World Trade Center, longitudinal study, cross-lagged analysis

## Abstract

Research on the longitudinal relationship between posttraumatic stress disorder (PTSD) and social support among survivors of large-scale trauma is limited. This study assessed bidirectional relationships between PTSD and perceived social support in a large sample of the 9/11-exposed cohort over a 14-year follow-up. We used data from 23,165 World Trade Center Health Registry (WTCHR) enrollees who were exposed to the 9/11 attacks and participated in the first four WTCHR surveys (Wave 1 (2003–2004) to Wave 4 (2015–2016)). PTSD symptoms were measured using the 17-item PTSD Checklist. Perceived social support was measured using the five-item version of the Modified Social Support Survey. We used a cross-lagged panel analysis and found an inverse relationship between PTSD symptoms and social support. PTSD at Wave 2 (W2) predicted less social support at Wave 3 (W3) (β = −0.10, *p* < 0.01), and PTSD at W3 predicted less social support at W4 (β = −0.05, *p* < 0.01). Conversely, social support at W3 buffered PTSD symptoms at W4 (β = −0.03, *p* < 0.05). Sub-analyses by types of perceived social support suggest greater effects of PTSD on emotional support than tangible support and in community members than rescue/recovery workers. Our findings suggest a bidirectional effect between PTSD symptoms and social support in a longitudinal study of 9/11-exposed populations.

## 1. Introduction

The terrorist attacks on the World Trade Center (WTC) on 11 September 2001 were unprecedented in mental health impact. Large-scale traumatic events, such as the 9/11 attacks, lead to an increase in posttraumatic stress disorder (PTSD). The prevalence of 9/11-related PTSD has varied from 3.8% to 29.6%, depending on populations and time periods [1]. Previous studies found that exposure to the 9/11 terrorist attack was associated with elevated PTSD levels among a cohort of WTC-exposed adults years after the disaster [2,3,4]. While the prevalence of PTSD among WTC survivors has decreased over time, elevated levels of PTSD have persisted for a substantial subgroup. For example, one study found that 17% developed PTSD during the first 13 years after 9/11, with half still showing symptoms of active PTSD 11–13 years later [5]. The burden of PTSD may vary according to the role the participant played in the WTC disaster and recovery [1].

PTSD is an impairing condition with long-lasting health effects [6]. Given the long-term health impact of PTSD, a growing body of research has focused on the role of social context as a potential protective factor against developing or worsening PTSD [7]. Social support is a time-varying multidimensional construct that reflects an individual’s resources for dealing with stress responses [8]. Two of the main subconstructs are perceived social support and received social support [9]. Received support refers to emotional, informational, or tangible assistance from families, friends, or coworkers [10]. Emotional support is embodied by behavior such as empathy, affection, and concern that allows an individual to know they are loved and valued [11,12]. By contrast, tangible support is behavior such as offering financial assistance, material goods, and services that directly address an individual’s needs [13]. Perceived social support refers to the support that is subjectively expected to be available to the individual when needed and may also reflect the individual’s satisfaction with the support [14]. Perceived social support could also be characterized by the types of support available (i.e., emotional vs. tangible) [15].

A better understanding of the relationship between PTSD and social support may be especially important in groups such as the WTC-exposed cohort, whose experiences affect their mental health years after the initial traumatic event. Currently, there are a limited number of long-term studies examining reciprocal relationships between social support and PTSD and social support after a large-scale collective traumatic event. The findings of previous studies on the relationship between social support and PTSD on WTC survivors have been varied. One study found that less perceived social support was associated with depression and not PTSD [16]. Another study found decreased social support was a risk factor for chronic PTSD among police responders to the WTC attacks [17]. A longitudinal study spanning 8–9 years reported that greater PTSD symptoms were associated with lower social support [18]. A separate study found that a PTSD symptom trajectory had a decreasing effect specifically on later emotional support [19]. Inconsistent findings from previous studies may result from a combination of factors including the form of trauma under investigation, the type of social support examined, the confounders included in the analysis, the study design, and the length of follow-up. More research is needed to understand the reciprocal relationship between PTSD and social support among the WTC-exposed population, especially whether differences in types of social support may have different relationships with PTSD.

Additionally, while PTSD is often associated with lack of social support, previous research has reported inconsistent results about the longitudinal relationship between these two variables. For example, some studies found that perceived social support does not predict subsequent PTSD symptoms [16,20,21]. In contrast, a recent meta-analysis of longitudinal studies reported a significant association in both directions [22]. One possible explanation for these seemingly conflicting results may be that the bidirectionality of the association between social support and PTSD may vary over time. For example, during the early stages post-trauma (e.g., before 12 months), poor social support is associated with greater PTSD symptom severity [23]. However, in later stages post-trauma (18–24 months), greater PTSD severity contributes to an erosion of social support [24].

Using longitudinal data collected by the World Trade Center Health Registry, we conducted this study to better understand the effect of the variable course of social support and PTSD. This analysis examines the time-varying relationship between PTSD symptoms and social support over 14 years among a cohort of 9/11-exposed individuals. The primary goal of this study was to determine whether there was a cross-lagged effect between PTSD and social support after controlling for prior levels of both variables. We also conducted sub-analyses to examine whether the cross-lagged relationship between PTSD and social support differed according to the type of perceived social support and whether the cross-lagged relationship differed by enrollee group. 

## 2. Materials and Methods

### 2.1. Study Population

This is a longitudinal study using information from the WTC Health Registry (“Registry”). The Registry enrolled over 71,000 individuals who lived, worked, or went to school in the area of the WTC disaster on 9/11 or who were involved in rescue and recovery efforts in 2003–2004 (Wave 1; W1) [25]. The Registry recruited participants through lists provided by employers, government agencies, and other entities (30%) and through an outreach campaign (70%). Data collected at enrollment (W1) included demographics, exposure incurred during and after 9/11, and health information, including PTSD symptoms. Since W1, the Registry has conducted four follow-up surveys in 2006–2007 (Wave 2, W2), 2011–2012 (Wave 3, W3), 2015–2016 (Wave 4, W4), and 2020–2021 (Wave 5, W5). At the time of the present study, we used data from W1 to W4 as W5 data was not available. We included participants who were aged 18 and over on 11 September 2001, and who had completed data from W1 to W4. We excluded participants with missing information on the self-reported PTSD Checklist (PCL) assessment at any wave and excluded participants with missing values on social support measures. The Centers for Disease Control and Prevention (CDC) and the NYC Department of Health and Mental Hygiene Institutional Review Boards approved the World Trade Center Health Registry protocol.

### 2.2. Social Support

Questions related to perceived social support were first asked at W3 and repeated at W4. We measured social support or perceptions of help from others using the five-item version of the Modified Social Support Survey (MSSS-5) [26]. MSSS-5 consists of five questions asking participants to rate on a five-point scale from none (0) to all of the time (4) whether they perceive someone was available when needed to take them to the doctor, have a good time with, hug them, prepare meals, and understand their problems. The MSSS-5 score ranged from 0 to 20. 

In addition, we divided the overall perceived social support into two types: emotional support (i.e., to have a good time with, to hug them, or to understand their problems), and tangible support (i.e., to take them to the doctor, or to prepare their meals) [19,27].

### 2.3. Exposure

The PTSD symptoms over four waves were measured using the PTSD Checklist-Specific (PCL-S). The 17 PCL-S items, specific to the events of 9/11, cover the three PTSD symptom clusters (re-experiencing, avoidance, hyperarousal) from the Diagnostic and Statistical Manual of Mental Disorders (Fourth Edition) criteria [28]. Enrollees self-reported whether they were bothered by symptoms in the past 30 days using a scale ranging from 1 (not at all) to 5 (extremely). We summed the response to a total severity measure with a range of 17 to 85. Previous research has validated PCL-S as a measure with good temporal stability, internal consistency (α > 0.75), test–retest reliability (correlation coefficient, r = 0.66), and high convergent validity (r = 0.58 to 0.93) [29]. 

### 2.4. Covariates

Covariates included as potential confounders in the multivariate analyses were sociodemographic characteristics (age, gender, race/ethnicity, education) at baseline and the Registry enrollee group (rescue/recovery workers vs. community members). Rescue/recovery workers included first responders, volunteers, and others who worked at the WTC site, debris loading sites, on barges, or at the Staten Island landfill any time during the 9-month rescue/recovery and cleaning efforts between 11 September 2001 and 30 June 2002. Community members include residents, children, and staff in schools (prekindergarten–12th grade) south of Canal Street and area workers and passersby south of Chambers Street at the time of the 9/11 attacks. Community members who were also rescue/recovery workers were grouped with rescue/recovery workers. In addition, race/ethnicity was grouped into non-Latino White vs. all others because previous studies using this dataset found non-Latino White enrollees had the lowest prevalence of probable PTSD among various racial/ethnic groups [2,25].

### 2.5. Statistical Analyses

We summarized demographic characteristics in the overall sample and among those with high baseline PTSD symptoms. We calculated descriptive statistics including mean (standard deviation, SD), Cronbach’s alpha internal consistency, and Pearson correlations for social support at W3 and W4, and PCL scores in all four waves. For descriptive analysis, SAS, version 9.4 (SAS Institute Inc., Cary, NC, USA) was used. A two-sided *p*-value < 0.05 indicated statistical significance.

We used a structural equation modeling approach to examine the reciprocal relations between PTSD and social support over 14 years, adjusting for age, gender, race and ethnicity, and educational attainment. Specifically, we conducted a cross-lagged longitudinal analysis [30,31] using the Lavaan package in R [32]. Cross-lagged regression coefficients reflect the directional effects of PTSD and social support on each other over this period. To examine potential differences in the association between social support and PTSD, we conducted sub-analyses stratified by enrollee group (rescue/recovery workers vs. community members) and by type of perceived social support (emotional vs. tangible).

## 3. Results

Among 71,426 exposed individuals enrolled at W1, 68,043 (95.3%) were 18 years or older on 9/11. Of the 68,043 enrollees, 27,959 participated in follow-up surveys W2 to W4. Compared to non-participants (*n* = 40,084) in any of the W2 to W4, participants in all three follow-up surveys were more likely to be male, non-Latino White, and to have higher educational attainment (Table S1). Participants were also slightly older at W1 (mean age 45.6 years (SD: 10.8) vs. 43.5 (12.8)) and had lower mean scores of PCL than non-participants (29.6 (SD: 12.4) vs. 31.5 (13.9)). 

Of the 27,959 enrollees who participated in all four waves, as mentioned above, we excluded those with incomplete data on social support (*n* = 1661) and PCL (*n* = 2948), and those with pre-9/11-diagnosed PTSD (*n* = 185), leaving a sample of 23,165 for the data analysis. Table 1 presents the characteristics of the overall study sample and the characteristics of those with high baseline PTSD symptoms (i.e., PCL ≥ 44). The sample was predominantly non-Latino White (75.6%) and male (62.5%). The overview of descriptive statistics of study variables is also provided in Table 2. The average scores of perceived social supports in both W3 and W4 were similar. In addition, the internal consistency for PCL at each wave was high (Cronbach’s alpha ranges from 0.93 at W1 to 0.95 at waves 2–4). The correlations between variables are shown in Table 3. We observed strong positive correlations between PCL scores and social support between waves. PCL autocorrelations between waves were above 0.70, and the social support autocorrelation between W3 and W4 was 0.68.

**Figure 1:**
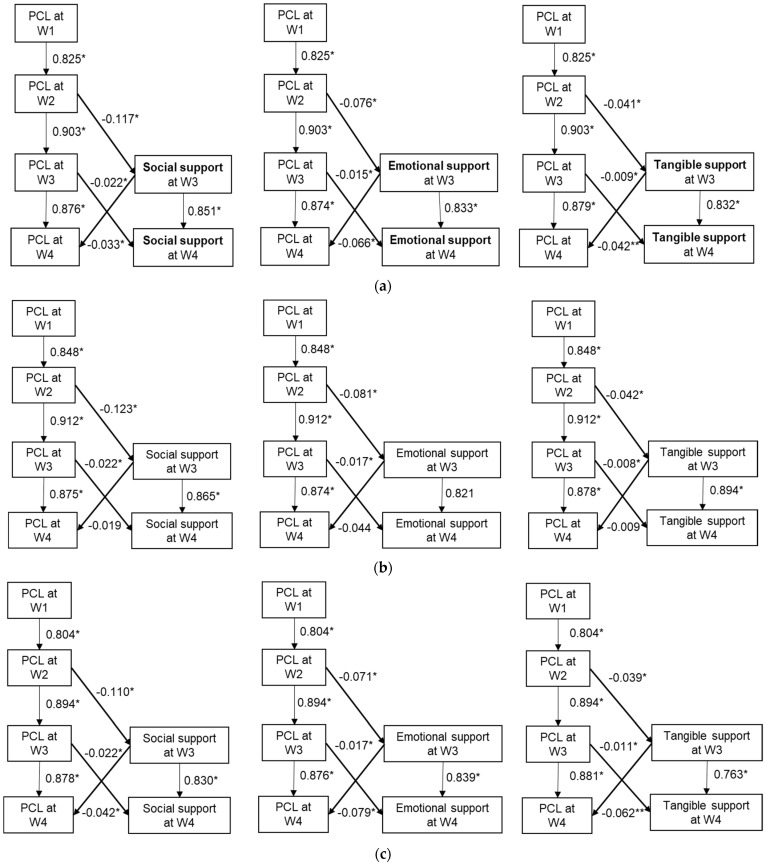
(**a**) Cross-lagged models for PTSD and social support through four waves in the full sample, WTCHR, 2003–2016 (*n* = 23,165) ^1^. (**b**) Cross-lagged models for PTSD and social support through four waves among rescue/recovery workers, WTCHR, 2003–2016 (*n* = 11,137) ^1^. (**c**) Cross-lagged models for PTSD and social support through four waves among community members, WTCHR, 2003–2016 (*n* = 12,028) ^1^. ^1^ Adjusted for age, gender, race/ethnicity, education. * *p* ≤ 0.001, ** *p* < 0.05.

Table 4 presents the model fit indices for model 1 to model 3. Figure 1a depicts the path coefficients for models 1–3. According to our cross-lagged analyses, PTSD at W2 predicted reduced social support at W3 (β = −0.117, 95% confidence interval (CI) = −0.122 to −0.112; *p* < 0.001), and PTSD at W3 predicted less social support at W4 (β = −0.022, 95% CI = −0.027 to −0.016; *p* < 0.001). However, we also found that social support at W3 buffered PTSD symptoms at W4 (β = −0.033, 95% CI = −0.052 to −0.014; *p* < 0.001) (Figure 1a). The regression coefficients for PTSD symptoms are larger for emotional support compared to tangible support. Figure 1b,c depict the path coefficients for models 1–3 stratified by type of enrollee. Similar to the overall population, PTSD symptoms were associated with lower social support in subsequent waves among rescue/recovery workers and community members. Perceived social support at W3 was associated with lower PTSD symptoms at W4 among community members only (β = −0.042, 95% CI = −0.066 to −0.017; *p* = 0.001). Although not statistically significant, the estimate for perceived social support at W3 was related to lower PTSD symptoms at W4 among rescue/recovery workers (β = −0.019, 95% CI = −0.048 to 0.011; *p* = 0.222).

## 4. Discussion

Our results indicate that previous waves’ PTSD symptom scores were highly associated with PTSD symptoms measured in subsequent waves. We found the same temporal pattern for social support. In addition, we found evidence of a bidirectional effect between PTSD and social support. Greater PTSD symptoms at a given wave predicted statistically significant lower social support in the subsequent wave. The regression coefficients suggest that the relationship between PTSD and social support may decrease over time. Sub-analyses by types of perceived social support suggest greater effects of PTSD on emotional support than tangible support and in community members than rescue/recovery workers. In addition, we found that social support buffered PTSD symptoms over time, especially among community members. The nonsignificant finding in the path of social support at W3 to the PCL at W4 among rescue/recovery workers may partly be due to their lower level of PTSD. Future analysis with more years of data in rescue/recovery workers is warranted to further our understanding of the bidirectional relationship between posttraumatic stress symptoms and social support.

Our results suggest that both the social erosion and social causation processes may be simultaneously occurring. Our finding of an inverse association between PTSD symptoms and subsequent social support is similar to previous longitudinal research on WTC survivors, supporting the erosion model [19]. Under the erosion model, individuals with PTSD symptoms have interpersonal problems leading to diminished social support over time [24]. Simultaneously, our observed findings of an inverse association between social support at W3 and PTSD symptoms at W4 also support the social causation model. The social causation model hypothesizes that social support buffers against PTSD by influencing an individual’s response to stressful encounters [33]. This finding is in line with most previous studies which report that increased perceived social support following a traumatic event is associated with decreased PTSD symptoms [34,35,36]. However, further research is needed to understand the psychological and biological mechanisms underlying the bidirectional effects.

Our other finding, that effects of PTSD may be larger for emotional social support than tangible support, may suggest one mechanism causing the bidirectional effects of social support and PTSD. Perceived emotional social support is well-documented to be an effective buffer against other mental health conditions such as depression [37,38]. Previous studies have suggested that healthy coping strategies may be one behavioral mechanism linking emotional support with positive mental health outcomes [39]. The relationship between tangible support and PTSD symptoms may be weaker than emotional social support because there may be negative consequences with receipt of tangible support. For example, one study found that individuals who received tangible support felt it diminished their sense of autonomy and caused feelings of helplessness [40]. Further research is needed to understand the utilization of different types of social support in relation to PTSD over time. In addition, future research on the bidirectional effects of PTSD and social support may want to consider whether effects differ according to severity of PTSD. The mechanisms linking PTSD and social support may vary depending on PTSD severity.

This study had several important strengths. It uses a unique, longitudinal dataset that follows a large cohort exposed to an unprecedented traumatic event for 14 years. The longitudinal design reduces selection bias and the large sample size improved study power and enabled us to examine subtypes of social support. The PTSD and social support measures were assessed at multiple times over a long follow-up period.

Despite these strengths, this study also had several limitations. First, the present study relied on self-reported PTSD symptoms and perceived social support, which might be affected by reporting biases or variability within individuals. However, the same measurements of PTSD and social support have been used across all surveys, and the instrument has been widely validated in population surveys or populations with traumatic exposure [26,29]. Second, the WTC Health Registry did not collect social support data at baseline. Moreover, the social support measures that were collected at W3 and W4 are a shorter version of the MOS Social Support Survey (MOS-SSS) [26]. Our study findings may underestimate the social support due to these data limitations. However, underestimation of social support would have biased our findings toward the null and so our study results are conservative estimates. Moreover, it remains unclear whether a measure of actually-received support would manifest the same pattern of results. Finally, the relatively low percent of PTSD among rescue/recovery workers may have resulted in the decrease in study power to detect the inverse association between social support at W3 and PTSD symptoms at W4. 

## 5. Conclusions

Finally, our results have important clinical implications. The long-standing relationship between PTSD and social support and the association between social support and PTSD suggest that PTSD treatment should include a component on the role of social support. A systematic review reported that social support plays an important role in the efficacy of cognitive-behavioral therapy for PTSD [41]. One study specifically found that social support moderated the association between PTSD treatment and change in PTSD symptoms [42]. Research has already suggested that post-disaster programs help survivors maintain their social support ties [43]. Our findings extend this recommendation of highlighting social support for clinicians dealing with survivors of large-scale collective trauma who may have chronic PTSD symptoms.

## Figures and Tables

**Table 1:** Characteristics of study sample at Wave 1 (*n* = 23,165).

	Total		
	No.	(%) ^1^	PCL ≥ 44 at Wave 1, %
Age, years			
18–44	11,139	(48.1)	11.7
45–64	11,271	(48.7)	13.2
≥65	755	(3.3)	6.5
Gender			
Male	14,483	(62.5)	9.7
Female	8682	(37.5)	16.4
Race/ethnicity			
Non-Latino White	17,510	(75.6)	9.5
All others	5655	(24.4)	20.7
Educational attainment			
Below college/unknown	9839	(42.5)	16.0
College or above	13,326	(57.5)	9.4
Enrollee group			
Rescue/recovery workers	11,137	(48.1)	9.7
Community members	12,028	(51.9)	14.6

^1^ May not sum up to 100% because of missing values.

**Table 2:** Descriptive statistics for primary variables (*n* = 23,165).

Variable	Mean	SD	Cronbach’s Alpha	Items	Range
PCL at Wave 1	28.99	11.97	0.93	17	17–85
PCL at Wave 2	30.89	13.64	0.95	17	17–85
PCL at Wave 3	30.04	13.30	0.95	17	17–85
PCL at Wave 4	28.52	12.62	0.95	17	17–85
Social Support at Wave 3	14.47	5.35	0.91	5	0–20
Social Support at Wave 4	14.50	5.16	0.91	5	0–20

**Table 3:** Correlation of study primary variables.

	PCL	PCL	PCL	PCL	Social Support
	at Wave 1	at Wave 2	at Wave 3	at Wave 4	at Wave 3
PCL at Wave 1	1.00				
PCL at Wave 2	0.71	1.00			
PCL at Wave 3	0.66	0.76	1.00		
PCL at Wave 4	0.61	0.72	0.79	1.00	
Social Support at Wave 3	−0.28	−0.31	−0.34	−0.29	1.00
Social Support at Wave 4	−0.29	−0.32	−0.33	−0.37	0.68

**Table 4:** Goodness-of-fit statistics for the tested models.

	Model 1	Model 2	Model 3
	(Social Support)	(Emotional Support)	(Tangible Support)
Chi-square	83,486.080	82,838.410	79,484.359
DF	40	40	40
*p*-value	<0.001	<0.001	<0.001
CFI	0.992	0.99	0.996
TLI	0.892	0.864	0.945
RMSEA	0.098	0.11	0.069
Sample size	23,165	23,165	23,165

## Data Availability

World Trade Center Health Registry data may be made available following review of applications to the Registry from external researchers.

## Supplementary Materials

The following supporting information can be downloaded at https://www.mdpi.com/article/10.3390/ijerph19052604/s1. Table S1: Comparison of participants of all four waves and non-participants in any of Waves 2–4.
