# Supplementary material for: The Bidirectional Relationship between Posttraumatic Stress Symptoms and Social Support in a 9/11-Exposed Cohort: A Longitudinal Cross-Lagged Analysis
Source: Int J Environ Res Public Health. 2022 Feb 24;19(5):2604. doi: 10.3390/ijerph19052604 (PMC8910094; doi:10.3390/ijerph19052604)
Supplement: Supplementary file 1 [file ijerph-19-02604-s001.zip › ijerph-1560687-supplementary.pdf]

Table S1. Comparison of participants of all four waves and non-participants in any of subsequent follow up Waves 2–4 [1].

	Participants (N = 27959)		Non-Participants (N = 40084)	
**Characteristic at Wave 1**	**No.**	**(%)**	**No.**	**(%)**
Age, years				
18-24	526	(1.9)	1472	(3.7)
25-44	12369	(44.2)	21572	(53.8)
45-64	13942	(49.9)	14600	(36.4)
≥65	1122	(4.0)	2440	(6.1)
Age, mean (SD)	45.60 (10.8)		43.5 (12.8)	
Gender				
Male	17169	(61.5)	23963	(59.8)
Female	10763	(38.5)	16121	(40.2)
Race/ethnicity				
Non-Latino White	20523	(73.4)	22860	(57.0)
All others	7436	(26.6)	17224	(43.0)
Educational attainment				
Below college/unknown	12471	(44.6)	21464	(53.5)
College or above	15488	(55.4)	18620	(46.5)
Enrollee group				
Rescue/recovery workers	13302	(47.6)	17196	(42.9)
Community members	14657	(52.4)	22888	(57.1)
PCL ≥44 scores				
Yes	3681	(13.2)	7003	(17.5)
No	23674	(84.7)	30970	(77.3)
Missing	604	(2.2)	2111	(5.3)
PCL, mean (SD)	29.6 (12.4)		31.5 (13.9)	

[1] Limited to those aged ≥18 on 9/11/2001.
